# Microstructural Evolution, Hardness, and Strengthening Mechanisms in SLM AlSi10Mg Alloy Subjected to Equal-Channel Angular Pressing (ECAP)

**DOI:** 10.3390/ma14247598

**Published:** 2021-12-10

**Authors:** Przemysław Snopiński, Anna Woźniak, Marek Pagáč

**Affiliations:** 1 Department of Materials Engineering and Biomaterials, Faculty of Mechanical Engineering, Silesian University of Technology, Konarskiego 18A Street, 44-100 Gliwice, Poland; 2 Materials Research Laboratory, Silesian University of Technology, Konarskiego 18A Street, 44-100 Gliwice, Poland; anna.wozniak@polsl.pl; 3 Department of Machining, Faculty of Mechanical Engineering, Technical University of Ostrava, 708 00 Ostrava, Czech Republic; marek.pagac@vsb.cz

**Keywords:** selective laser melting, ECAP, grain refinement, EBSD, microstructure, hardness

## Abstract

The AlSi10Mg alloy is characterized by a high strength-to-weight ratio, good formability, and satisfying corrosion resistance; thus, it is very often used in automotive and aerospace applications. However, the main limitation of using this alloy is its low yield strength and ductility. The equal-channel angular pressing is a processing tool that allows one to obtain ultrafine-grained or nanomaterials, with exceptional mechanical and physical properties. The purpose of the paper was to analyze the influence of the ECAP process on the structure and hardness of the AlSi10Mg alloy, obtained by the selective laser melting process. Four types of samples were examined: as-fabricated, heat-treated, and subjected to one and two ECAP passes. The microstructure analysis was performed using light and electron microscope systems (scanning electron microscope and transmission electron microscope). To evaluate the effect of ECAP on the mechanical properties, hardness measurements were performed. We found that the samples that underwent the ECAP process were characterized by a higher hardness than the heat-treated sample. It was also found that the ECAP processing promoted the formation of structures with semicircular patterns and multiple melt pool boundaries with a mean grain size of 0.24 μm.

## 1. Introduction

A great deal of data from the literature highlight the fact that the SLM process allows the fabrication of complex geometries, which is favorable for custom-made parts. Design freedom with optimized geometries, reduced production steps, reduced cost and time, and the possibility of recycling of waste material are well-known advantages of additive manufacturing technologies [1]. However, currently, it is beginning to become apparent that the SLM technology not only facilitates the production process, but also guarantees significant changes in the structure that are necessary to optimize the properties of the final elements. In fact, the greatest advantage offered by the SLM method is the possibility of obtaining samples with a peculiar microstructure caused by thermal gradients and non-equilibrium solidification during the process [2,3]. The microstructural evolution is determined by the parameters of the SLM process. The power of the laser beam, its size, and scan speed determine the melt pool geometry, which has a significant effect on the solidification kinetics. Then, thermal cycling and cooldown determine future precipitation kinetics, phases, and grain growth. Interestingly, the geometry of the fabricated part can affect local heat transfer conditions and, in effect, can affect solidification, defects, and microstructure—small elements will reach a higher temperature during melting as compared to larger parts, given constant power and speed [4,5]. This can provide for more defects in smaller parts of the detailed geometry. However, the fine-grained structure of the SLM elements guarantees their enhanced mechanical strength and provides suitable starting points for severe grain refinement. This phenomenon is caused by the elevated amount of grain boundaries that act as obstacles to moving dislocations [6]. Furthermore, the 3D-printed AlSi10Mg alloy has an ultrafine cellular microstructure composed of the soft Al ‘core’ and the interconnected Si ‘shell’, which is generally characterized by outstanding hardening ability [7]. This is because the mechanical incompatibility between Al and Si leads to the accumulation of geometrically necessary dislocations (GND) during strain hardening [8]. The existing microstructural gradient (soft Al/hard Si) is also believed to be an additional factor that can promote microstructural refinement, as GNDs provide a continuous increase in subgrain boundary misorientations [9]. Therefore, intuitively, one might expect that ECAP can be used successfully as a novel post-SLM processing tool for the improvement of mechanical properties to obtain ultrafine-grained/nanostructured materials with superior properties. This raises the question as to whether it is possible for processing–microstructure–property relationship widows to develop in order to intensify the grain refinement of SLM elements and to obtain better mechanical properties?

Among various series of SLM aluminum alloys, the AlSi10Mg alloy has gained particular attention in the materials science community. This is because this alloy exhibits high specific strength, good corrosion resistance, and excellent weldability [10]. Due to these excellent properties, the AlSi10Mg alloy can be used in the automobile, aerospace, automotive, and marine industries. However, the yield strength (YS) of the AlSi10Mg alloy in as-built condition is lower than 290 MPa and its ductility is only about 5–10%, respectively. Such mechanical properties cannot meet the requirements of some medium- and high-strength structural parts in the aerospace and automotive fields. Therefore, to further increase the mechanical properties, post-processing treatment is usually employed.

To date, many investigators have tried to strengthen the AlSi10Mg alloy by heat treatment to peak strength (i.e., artificial T6 aging or direct aging). However, according to Aboulkhair et al. [11], a typical precipitation treatment resulted in the softening of SLM AlSi10Mg. However, natural aging was found to be an effective way to enhance the mechanical properties of the SLM AlSi10Mg alloy. As demonstrated by Park et al. [12], direct aging treatment resulted in an increase in (YS) to 310 MPa; unfortunately, the improvement in mechanical properties was achieved at the expense of elongation, which decreased to 6.2%.

Another strategy aimed at improving the mechanical properties of the AlSi10Mg alloy of LPBF is the addition of reinforcement particles to Al-Si-based matrices to produce composite materials using SLM [13,14]. In this context, Li et al. [15] conducted excellent work, producing TiB_2_/AlSi10Mg composite parts with a superior tensile strength of 539 MPa and an excellent elongation of 15.5%. In addition, other researchers introduced some hard ceramic particles such as SiC [16], Al_2_O_3_ [17] and TiN [18] into the aluminum powder using a high-energy ball milling method. However, it is important to note that in situ synthesis and spray deposition are time-consuming and expensive, whereas a high-energy ball milling method could reduce powder sphericity, thus causing poor processing performance.

Other post-processing treatments include hot isostatic pressing (HIP) [19,20] or combination of HIP with a subsequent T6 treatment [21]. Although these post-processing treatments are effective in reducing inner imperfections, such as unmolten areas or bonding errors between the borders of the melt pool and the pores, the mechanical properties of aluminum alloys of SLM, such as AlSi10Mg, AlSi12 and AlSi20, were reported to decrease, since the HIP process is conducted at elevated temperature [22].

A niche approach used to enhance the mechanical properties of LPBF aluminum alloys is severe plastic deformation. To date, only a few investigators have examined the effect of high-pressure torsion (HPT) [23] and equal channel angular pressing (ECAP) [24] on the microstructure and mechanical properties of LPBF Al-Si alloys. It was established that high-pressure torsion can substantially reduce the pore diameter even at low strain levels, and significantly increase the microhardness of the AlSi10Mg alloy. Similar behavior was shown for the AlSi12 alloy subjected to ECAP. Furthermore, the later work of Hosseinzadeh et al. [24] on the AlSi12 alloy confirmed a beneficial effect of ECAP on the mechanical properties of LPBF Al-Si alloy. They showed that the ultimate tensile strength, the yield strength, and the ductility of the selectively laser-melted AlSi12 alloy were improved by 56%, 11%, and 55% after four passes of ECAP, respectively.

Meanwhile, it is only very recently that the microstructure and mechanical properties of SLM-fabricated and then ECAP-processed AlSi10Mg alloy were investigated by the current authors [25]. In this article, it was found that the yield strength increased significantly (up to 382 MPa). However, the reasons behind the observed high yield strength, particularly grain refinement, were not studied in detail.

Therefore, this article aims to extend the previous research and further investigate the microstructural evolution using electron backscatter diffraction (EBSD) and transmission electron microscopy, as well as to elucidate the main strengthening mechanisms that contribute to the yield strength of the AlSi10Mg alloy processed by ECAP.

## 2. Materials and Methods

In this work, samples were made using the selective laser melting (SLM) method. We used AlSi10Mg alloy spherical powder prepared by the aerosol method. Table 1 gives its nominal chemical composition.

**Figure 1:**
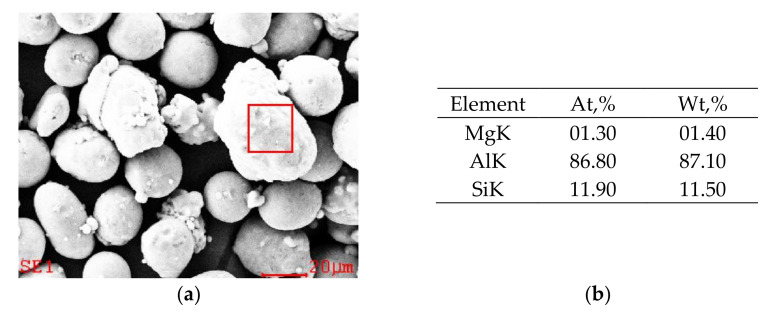
Examples of results of SEM observation: (**a**) AlSi10Mg alloy powder. (**b**) The chemical composition estimated by EDS analysis.

Figure 1a shows the secondary electron (SEM) image of the AlSi10Mg alloy powder used in this investigation. As seen, the powder particles were spherical with a mean size of 25 μm. The chemical composition estimated by EDS analysis of AlSi10Mg powder is shown in Figure 1b.

### 2.1. SLM Process

**Figure 2:**
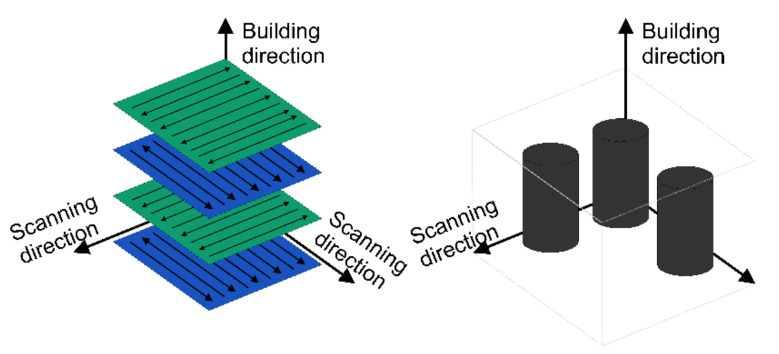
Visualization of the applied scanning strategy.

The AlSi10Mg alloy samples were fabricated on a commercial SLM device, namely the TruPrint 1000 system from Trumpf (Germany). The builds were fabricated in an argon environment with constant oxygen level <0.5%. The preparation of the process, i.e., the slicing of the model, the scanning strategy, and the orientation of the components, were designed using Materialise Magics software. The bidirectional scanning strategy (Zig-Zag), Figure 2, was used as the scanning strategy to decrease the thermal stress between layers, while the scanning angle was alternated by 90° upon the precedent layer. The main SLM process parameters used for sample fabrication are given in Table 2.

### 2.2. Heat Treatment

Because the as-built (SLM) samples were rather brittle, it was necessary to perform a heat treatment prior to the ECAP process. For this purpose, the as-built SLM samples were heat treated in a laboratory dryer under an argon atmosphere. The parameters of the heat treatment were as follows:

- Time—8 min;
- Temperature—300 °C.

This heat treatment allowed partial preservation of the unique cellular microstructure composed of a hard Si ‘shell’ and a soft Al ‘core’. As discussed in the Introduction section, such a cellular (heterogeneous) microstructure offered outstanding strain hardenability; however, their partial disintegration resulted in the decrement of the alloy hardness, which is discussed later. A more detailed analysis of the decomposition of the cellular substructure after low-temperature annealing was discussed in our previous article [25].

### 2.3. ECAP Process

Before the ECAP process, cylindrical rods were machined to a diameter of 9.95 mm. The samples were then lubricated using MoS_2_ and pressed through a 120° ECAP die (introducing an equivalent strain of ε = 0.6 in each pass) through route A (without rotation between consecutive passes) up to two pressings. We prepared three samples for each condition.

### 2.4. Porosity and Microstructural Characterization

The density of the tested samples was measured using the Archimedes method. Analytical Radwag balance AS220.R2 type (±0.1 mg) was used. Before the density test, the samples were placed in an ultrasonic bath for 15 min (ethyl alcohol and acetone 2:1). Measurements were repeated five times. The density was calculated according to Formula (1), indicating the measurement of the mass of the samples in air and fluid (deionized water); *ρ_fl_*—density of the deionized water, 0.998203 g/cm^3^ at T = 20 ± 1 °C; *ρ_a_*—density of the air, 0.001205 at T = 20 ± 1 °C; *m_a_*—mass of the samples in air; *m_f_*—mass of the samples in deionized water.

(1) $$\rho_{c}=\frac{m_{p}}{m_{p}-m_{c}}\left(\rho_{w}-\rho_{p}\right)+\rho_{p}$$

For Archimedes density measurements, we used three different samples for each tested condition.

ImageJ analysis software was used to characterize defects in as-received, heat-treated and ECAP-processed AlSi10Mg aluminum alloy samples from optical micrographs obtained during light microscopy observations. Five images were used for each sample (as received, heat-treated, 1 ECAP and 2 ECAP). Firstly, the images were binarized, i.e., converted into black and white using a pre-set threshold value, in which black corresponded to porosity, while white was the fabricated sample. The percentage of volume (Vol.%) of pores in the samples could be obtained by calculating the ratio of black to white pixels.

The microstructures of the samples were characterized using an inverted Axio Observer Z1 light microscope (LM) and a Zeiss Supra 35 scanning electron microscope. For this purpose, the as-built, heat-treated, and ECAP-processed samples were cross-sectioned, grinded using SiC papers, and polished using a 0.04 µm colloidal silica. The polished surfaces were then etched using Keller’s reagent.

Characterization of microstructure via EBSD was conducted on a ZEISS Supra 25 scanning electron microscope, with a step size of 300 and 60 nm for the as-built and ECAP-processed sample, respectively.

TEM examination was carried out on a microscope (JEM 3010UHR, JEOL) operating at an accelerating voltage of 200 kV. For the TEM characterization, a 0.5-mm disc was sectioned from the center of the cross section of the ECAP-processed sample (ND-TD plane). The sample was then electrolytically polished using an A2 Struers polishing solution and the TENUPOL polishing unit. The following electropolishing process parameters were used:

- Temperature of polishing solution—30°;
- Polishing solution flow rate = 12;
- Voltage = 20 V.

### 2.5. Hardness

**Figure 3:**
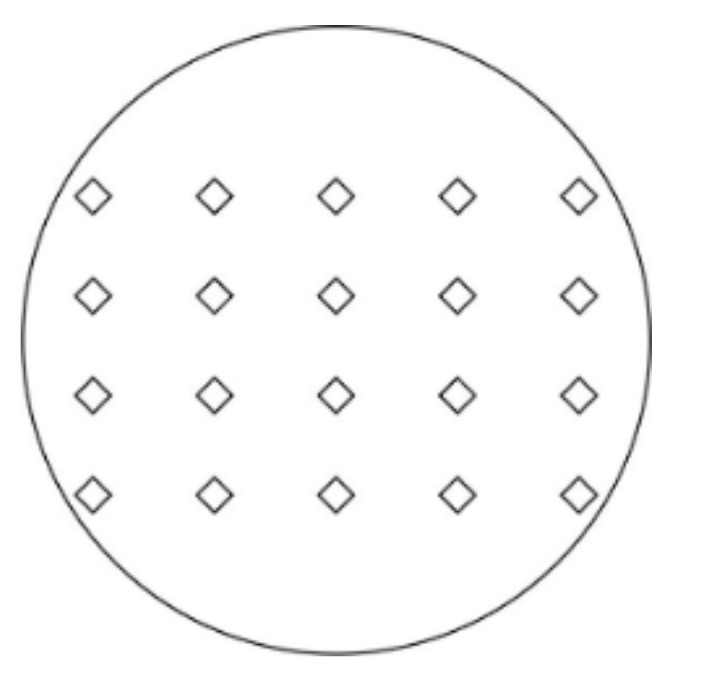
Indentation matrix used in this article to investigate hardness distribution.

Vickers microhardness (HV) tests were conducted on the cross-section plane of the as-built, heat-treated and ECAP-processed samples using a Future-Tech microhardness tester under a load of 100 gf with a dwell time of 15 s. A total of 20 individual indentations were made according to the indentation matrix (Figure 3).

## 3. Results

### 3.1. Effects of Heat Treatment and ECAP on Porosity

The results of the density measurements obtained by the Archimedes method are presented in Table 3. On the basis of this, it can be concluded that the ECAP process provided a higher density value, in comparison to the value obtained for as-building samples and samples after the heat-treatment process.

The sample subjected to two passes of ECAP was characterized by the highest density, and the mean value was 2.65 ± 0.04 g/cm^3^, which constituted approximately 99.5% of the theoretical density value (ρ = 2.67 g/cm^3^).

**Figure 4:**
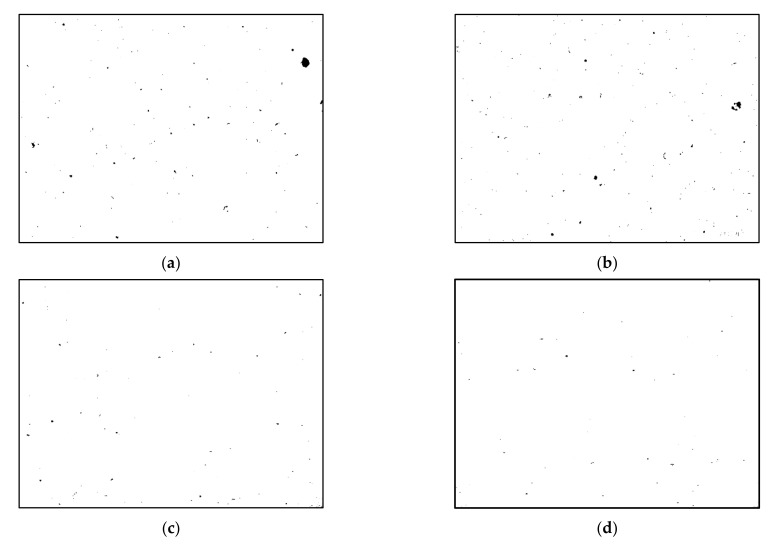
Porosity analysis after image processing (binarized images): (**a**) as-built, (**b**) heat-treated, (**c**) heat-treated + 1 ECAP pass, (**d**) heat-treated + 2 ECAP passes.

Figure 4 shows binarized images of the as-built, heat-treated, 1 ECAP, and 2 ECAP samples, respectively. In this figure, the visible black areas correspond to the porosity on the tested plane. As shown in Figure 4a,b, the mean porosity contents for the as-built and heat-treated samples were similar. The measured porosity levels were 0.25% and 0.24% for the as-built and heat-treated samples, respectively. A remarkable decrease in defects was revealed in the severely deformed samples. After the first and second ECAP passes, the porosity (defects) levels decreased to 0.09% and 0.05%, respectively. The results confirm that even a relatively small number of ECAP pressings is capable of effectively reducing porosity in additively manufactured parts.

### 3.2. Microstructure

#### 3.2.1. Light Microscopy

**Figure 5:**
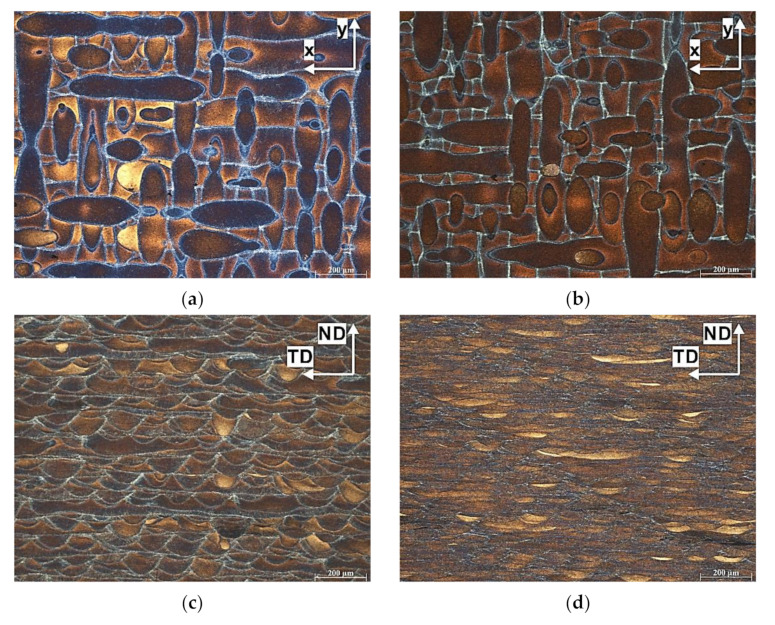
Microstructure of the AlSi10Mg alloy: (**a**) as-built, (**b**) heat-treated, (**c**) heat-treated + 1 ECAP pass, (**d**) heat-treated + 2 ECAP passes.

Figure 5a shows the microstructure of the as-built S0 sample taken in the horizontal cross section. Etching with Keller’s reagent revealed long laser scan tracks; thus, the scanning strategy and the hatch distance could be determined directly from this micrograph. As can be seen, an alternating bidirectional chessboard strategy (Zig-Zag) with a 90° rotation between contiguous layers was applied.

Figure 5b shows the microstructure of the heat-treated sample. Although this sample was heat treated, light microscopy observation did not reveal any significant change in microstructure with respect to the as-built sample.

Figure 5c,d show the optical micrographs of the ECAP-processed samples. A characteristic microstructure consisting of multiple semicircular patterns was created in the sample subjected to one pass of ECAP (Figure 5c). The microstructure evolved progressively after the second ECAP pass. The semicircular patterns became compressed and elongated along the x axis (Figure 5d), and thus, their height decreased from 54 ± 6 μm (1 ECAP pass) to 16 ± 5 μm (2 ECAP passes). To clarify the observed microstructural evolution, it is necessary to refer to the theoretical shearing patterns during ECAP. According to Furukawa et al. [26], in route A, the macroscale shear deformation is vertical to the intersection plane, which is the same direction as the tangent of the flow lines. During ECAP, the work sample experiences compression along the extrusion direction (ED) and tension in the transverse direction (TD). This is usually revealed as a series of parallel macroscopic bands on the ND-TD plane. As shown, our results are in line with Furukawa’s work since chemical etching revealed the formation of the layered/elongated structure.

#### 3.2.2. EBSD

**Figure 6:**
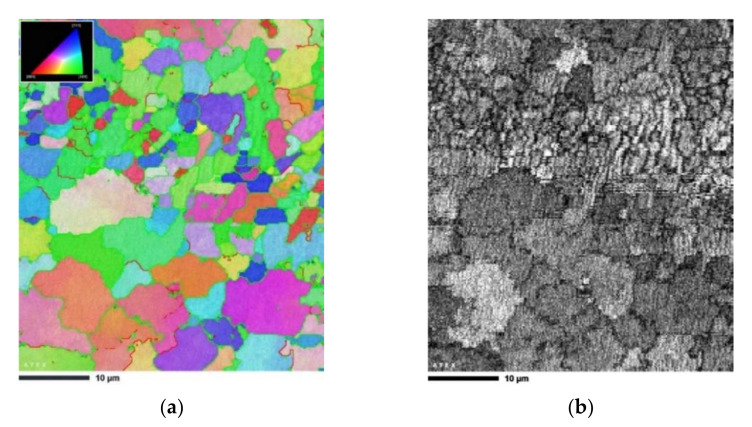
EBSD micrograph of the heat-treated sample: (**a**) IPF-Z map, (**b**) image quality (IQ) map. The standard triangles shown to the upper left of IPF map refer to color coding.

Figure 6 shows the EBSD mapping results of the heat-treated sample in the XY cross section. In the analyzed area, the average grain size was approximately 3.5 µm with a relatively high standard deviation of 2.1 µm. This was the case because a bimodal microstructure, consisting of fine and larger equiaxed morphologies, was created in the material. As seen in Figure 6a, the laser scan track boundary consisted of fine grains (approximately 1–2 µm in size) with an equiaxed morphology, whereas the grains in the laser scan track interiors were characterized by a larger size (approximately 8–10 µm in size).

**Figure 7:**
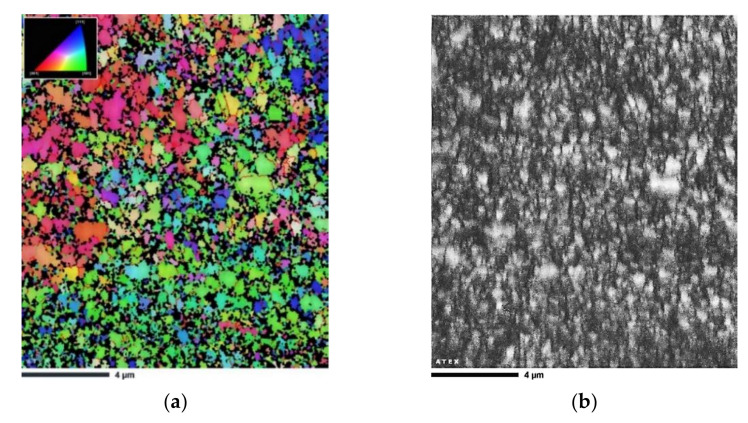
EBSD micrograph of the heat-treated sample subjected to 2 ECAP passes: (**a**) IPF-Z map, (**b**) image quality (IQ) map. The standard triangles shown to the upper left of IPF map refer to color coding.

A significant grain refinement occurred after two passes of ECAP (Figure 7). The bimodal microstructure of the heat-treated AlSi10Mg alloy was modified to become ultrafine-grained. The grain size of the AlSi10Mg alloy was reduced to 240 nm with a standard deviation of 180 nm. This grain refinement was attributed to the unique microstructural evolution of the investigated XY plane, an increased fraction of melt pool borders consisting of fine equiaxed grains (see Figure 5d), as well as simple shear deformation and the accumulation of a high density of dislocations.

#### 3.2.3. TEM

**Figure 8:**
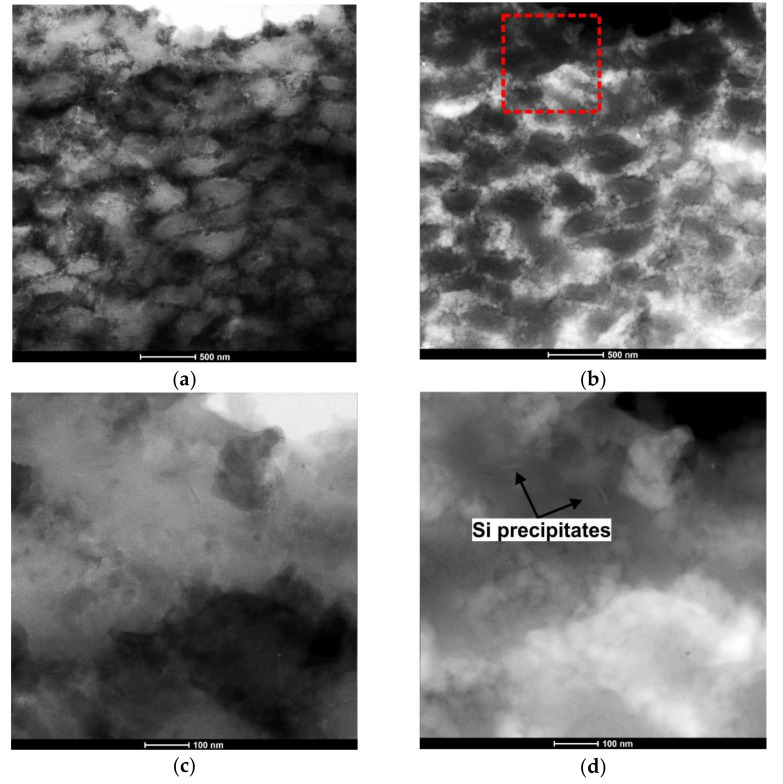
Microstructure of the AlSi10Mg alloy after two passes of ECAP: (**a**) STEM image obtained from the XY section, (**b**) HAADF STEM image obtained from the XY plane, (**c**,**d**) STEM and HAADF STEM images obtained from the red area in (**b**).

Figure 8 shows the microstructure of the sample subjected to two passes of ECAP. Observation at higher magnification using TEM revealed multiple cellular structures with a mean size in the range of 300–400 nm. In the STEM image (Figure 8a), the darker areas correspond to the heavily defected zones and Si subgrains with a high dislocation density. The bright field and the HAADF image pairs associated with the red area, illustrated by a square in Figure 8b, revealed Si precipitates with diameters of between 60 and 80 nm distributed within a primary α-Al phase.

**Figure 9:**
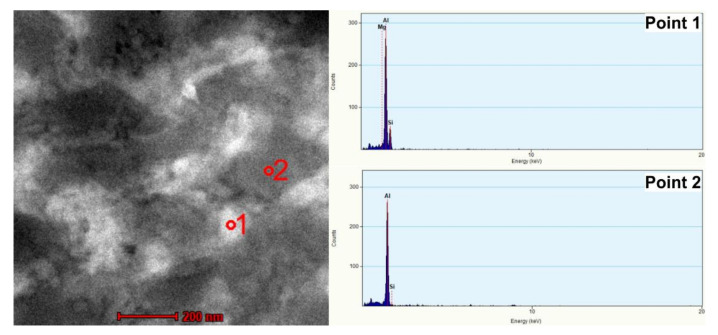
BF-STEM micrograph and EDS spectra of point 1 and point 2.

**Figure 10:**
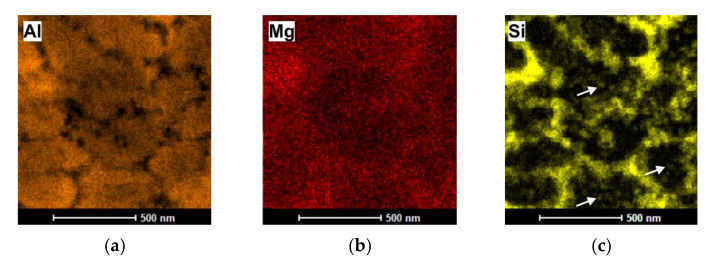
EDS elemental maps of Al, Si, and Mg (AlSi10Mg alloy after two passes of ECAP): (**a**) Al, (**b**) Mg, (**c**) Si.

To analyze the chemical composition of the cellular structures, energy-dispersive X-ray spectroscopy (EDS), in the scanning mode of transmission electron microscopy (TEM), scanning TEM (STEM), was used and the results are presented in Figure 9 and Table 4. As implied by the EDS measurement, the cell interior was an α-Al phase, whereas the cell boundaries were decorated with an interrupted Si network (with a quasi-eutectic composition of the casting alloy). The EDS elemental map (Figure 10) confirmed the presence of nanosized Si precipitates distributed within the α-Al phase (see white arrows).

### 3.3. Hardness Distribution

**Figure 11:**
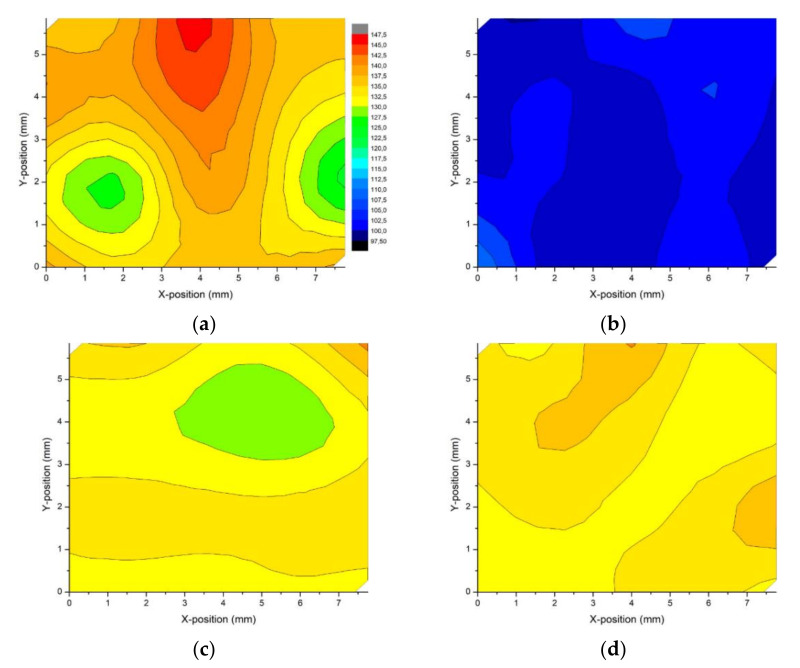
Microhardness distribution throughout the sample: (**a**) as-built, (**b**) heat-treated, (**c**) 1 ECAP pass, (**d**) 2 ECAP passes.

Figure 11 shows color-coded contour maps that were generated based on micro-hardness measurements according to the indentation matrix in order to visualize the micro-hardness distribution throughout the sample surface. As seen in Figure 11a, the highest average microhardness value of 135 HV was recorded for the as-built sample, but the hardness distribution was inhomogeneous. Heat treatment resulted in the decrement of the microhardness to 101 HV. In addition, the degree of hardness inhomogeneity was weakened compared with that of the as-built sample.

The first ECAP pass introduced multiple dislocations into the material; therefore, the microhardness of the heat-treated AlSi10Mg alloy increased from 101 to 132 HV. Along with microstructural refinement (second ECAP pass), there was only a minor increase in microhardness to 133 HV, which indicated that hardness saturation was achieved.

Taking into account metallographic observations, the registered deviation in the microhardness value (hardness inhomogeneity) was the result of the unique nonuniform microstructure of the SLM as-built sample. Additionally, the inhomogeneous distribution of pores might have caused a decrease in microhardness in the pore-rich regions. In addition, residual stresses might have affected a non-uniform hardness distribution. After heat treatment, the as-built sample was stress relieved; therefore, the hardness decreased and a more homogeneous hardness distribution was achieved. Further processing via ECAP caused a unique microstructural evolution and a decrease in porosity of the heat-treated sample. Therefore, the ECAP-processed samples exhibited a more uniform distribution of microhardness, with only minor variations caused by strain inhomogeneity.

### 3.4. Analysis of Strengthening Mechanisms of the ECAP-Processed Sample

Taking into account the microstructural characteristics of the severely deformed microstructure of the AlSi10Mg alloy, the strengthening mechanisms in the material can be attributed to the Orowan mechanism (owing to existence of nanosized Si precipitates distributed within the α-Al phase), the Hall–Petch effect (due to grain refinement), the solid solution (due to Si in the solid solution), and dislocation hardening. Therefore, the yield strength (*σ_y_*) can be calculated as follows:

(2) $$\sigma_{y}=\sigma_{0}+\Delta \sigma_{Orowan}+\Delta \sigma_{Hall-Petch}+\Delta \sigma_{Solid\;solution}+\Delta \sigma_{dislocation}$$

where *σ*_0_ is an internal friction stress (~72 MPa for Al) [27].

(3) $$\Delta \sigma_{Orowan}=\frac{\varphi Gb}{d_{Si}}{\left(\frac{6V_{Si}}{\pi }\right)}^{1/3}$$

where *φ* is a material constant (in the range of 0.15–0.4 [28]) estimated as 0.15 for coherent and 0.4 for semi-coherent Si precipitates, *G* is the shear modulus of the Al matrix (~26.5 GPa), *b* is the Burgers vector of the Al (~0.286 nm), *d_Si_* is the average diameter of the Si precipitates (taken as ~70 nm) and *V_Si_* is the volume fraction of the Si precipitates (~2.5 vol.%) [15]. The strength increase calculated via Orowan strengthening was 79 MPa.

(4) $$\Delta \sigma_{Hall-Petch}=\frac{k}{\sqrt{d}}$$

where *k* is a constant (~0.04 MPa·m^1/2^ for Al [29]) and d is the mean grain size (EBSD analysis). The yield strength increment estimated via grain size strengthening was equal to 81.6 MPa.

(5) $$\Delta \sigma_{Solid\;solution}=K_{Si}{\left(\omega_{Si}^{\alpha }\right)}^{m}$$

where $K_{Si}$ is 11 MPa wt.%^−1^. The exponent m, in general, is in the range from 0.5 to 1 and $\omega_{Si}^{\alpha }$ is the Si element concentration in at% (EDS analysis using TEM). The estimated yield strength increment due to the Si in the solid solution was equal to 13.2 MPa.

In conventional homogeneous materials, the strength increment caused by the increased density of dislocations is calculated using the following equation:

(6) $$\Delta \sigma_{dislocation}=\beta MGb\sqrt{\rho_{d}}$$

where *β* is a material constant (0.16), *M* is the Taylor factor (3.06) [30,31], *G* is the shear modulus (taken as 26 GPa), $\rho_{d}$ is the dislocation density 1.23 × 10^15^ m^−2^ (the dislocation density was taken from the XRD results published in our previous article [25]), and *b* is the Burgers vector of the Al (~0.286 nm).

However, in the case of additively manufactured alloys, the strength increment should be considered as a sum of statistically stored dislocations (SSDs) and geometrically necessary dislocations (GNDs) which is given by the following equation:

$$\rho_{d}=\rho_{SSD}+\rho_{GND}$$

**Figure 12:**
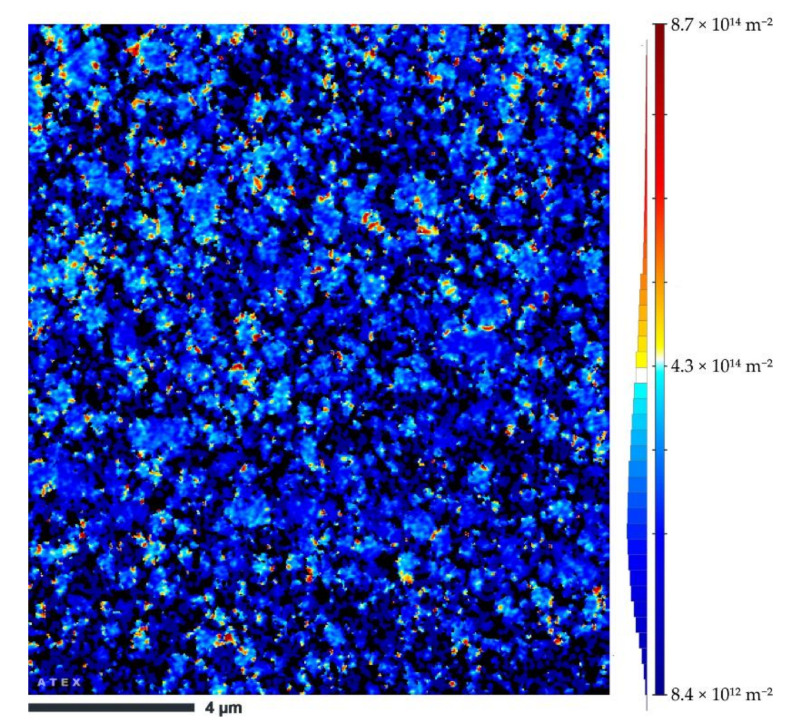
GNDs density distribution of the heat-treated + 2 ECAP pass sample.

The GNDs density can be calculated based on the EBSD results (based on the kernel average misorientation map), which is shown in Figure 12. Consequently, the density of GNDs in the sample subjected to two passes of ECAP was equal to 1.42 × 10^14^ m^−2^.

The estimated strength increment due to mutual geometrically necessary and statistically stored dislocation was 186.7 MPa.

Taking into account the strengthening components, the increases in strength in the sample after two passes of ECAP can be expressed as follows:

(7) $$\sigma_{y}=72_{friction\;stress}+79_{Orowan}+81.6_{Hall-Petch}+186.7_{dislocation}=419.3\;MPa$$

According to Equation (7), the calculated yield strength of the AlSi10Mg alloy subjected to two passes of ECAP was equal to 419.3 MPa. This estimated value of YS was similar to the experimental value of 382 MPa obtained from the static compression test in our previous study [25].

## 4. Conclusions

In this article, the effect of ECAP on the microstructure and hardness of selectively laser-melted AlSi10Mg alloy was investigated. The main conclusions are depicted as follows:

- The image analysis revealed that the ECAP led to a reduction in the porosity of the selectively laser-melted AlSi10Mg samples. It was found that the fraction of porosity defects decreased with an increase in the number of ECAP pressings.
- ECAP eliminated the pores of the SLM-AlSi10Mg alloy; therefore, the density increased from 2.51 to 2.65 g/cm^3^.
- Heat treatment had a negligible effect on the microstructure of the AlSi10Mg alloy. ECAP processing promoted the formation of a novel layered structure that comprised semi-circular patterns and multiple melt pool boundaries, whose fraction increased by increasing the number of ECAP pressings.
- The EBSD analysis revealed that the mean size of grains decreased from 3.5 μm to 0.24 μm after two pressings of ECAP.
- Heat treatment caused a decrease in the mean microhardness value from ~135 to ~101 HV. After ECAP processing, the hardness increased to ~133 HV.
- Based on the strengthening mechanism analysis, it can be concluded that the increment in the σ_y_ value of the AlSi10Mg alloy after ECAP resulted from the Orowan mechanism, the grain boundary (Hall–petch), the solid solution, and dislocation strengthening. The greatest contribution to the overall σ_y_ came from the exceptional grain refinement (grain boundary) and dislocation strengthening.

## Figures and Tables

**Table 1:** Nominal chemical composition of the AlSi10Mg alloy, wt.%.

Element, wt. (%)								
Si	Mg	Fe	Ti	Zn	Mn	Ni	Co	Al
9–11	0.25–0.45	<0.25	<0.15	<0.10	<0.10	<0.05	<0.05	Balance

**Table 2:** Overview of SLM process parameters.

Measured Laser Power, W	Layer Thickness, μm	Laser Beam Diameter, μm	Scan Speed, mm·s^−1^
175	20	55	1400

**Table 3:** Results of density measurements obtained by Archimedes methods.

Sample	As-Built	Heat-Treated	Heat-Treated + 1 ECAP Pass	Heat-Treated + 2 ECAP Passes
Density (g/cm^3^)	2.51 ± 0.18	2.52 ± 0.18	2.61 ± 0.04	2.65 ± 0.04

**Table 4:** Results of the EDS pointwise chemical composition microanalysis.

Point	Element	Wt, %	At, %
1	Al	82.8	83.2
	Si	15.9	15.4
	Mg	1.3	1.4
2	Al	98.4	98.5
	Si	1.2	1.1
	Mg	0.4	0.4

## Data Availability

Not applicable.
